# Succinate aggravates pulmonary fibrosis through the succinate/SUCNR1 axis

**DOI:** 10.1152/ajplung.00286.2024

**Published:** 2025-03-27

**Authors:** Rishi Rajesh, Agnes Anna Mooslechner, Hannah Schweighofer, Svetlana Pahernik, Ilse Lanz, Reham Atallah, Wolfgang Platzer, Clemens Aigner, Alberto Benazzo, Stefano Angiari, Leigh Marsh, Grazyna Kwapiszewska, Akos Heinemann, Thomas Bärnthaler

**Affiliations:** 1 Otto Loewi Research Center, Division of Pharmacology, Lung Research Cluster, Medical University of Graz, Austria; 2 Department of Thoracic Surgery, Medical University of Vienna, Vienna, Austria; 3 Otto Loewi Research Center, Division of Immunology, Medical University of Graz, Austria; 4 Otto Loewi Research Center, Lung Research Cluster, Graz, Austria; 5 Institute for Lung Health, Cardiopulmonary Institute, Member of German Lung Center ( DZL), Giessen, Germany; 6 Research Unit Molecular Pharmacology in Pulmonary Disease, Medical University of Graz, Austria

## Abstract

**Introduction:**

Idiopathic pulmonary fibrosis(IPF) is a chronic progressive lung disease that leads to destruction of alveoli and replacement by fibrotic tissue. Metabolic profiling of lung tissue and serum from IPF patients has revealed that levels of tricarboxylic acid (TCA) cycle metabolites such as succinate are altered in patients with IPF. In our study, we aim to evaluate the role of succinate and its receptor-succinate receptor 1 (SUCNR1) in the pathogenesis of lung fibrosis, with a focus on fibroblasts, a central cell in IPF.

**Methods:**

SUCNR1 expression was investigated using Western blots, qPCR, and FISH. In vitro assays with IPF and normal human lung fibroblasts(NHLF) were used to evaluate the effect of succinate treatment on the expression of fibrotic markers, fibroblast-myofibroblast transition, apoptosis and signaling mechanisms. Studies with the bleomycin mouse model of PF were used to evaluate the effect of succinate in vivo.

**Results:**

Several cell types in the lung express SUCNR1 including ATII cells, fibroblasts, and macrophages. In IPF patient fibroblasts, succinate treatment increased expression of fibrosis associated markers such as alpha smooth muscle actin and collagen. Moreover, succinate exaggerated TGF-β-mediated fibroblast-to-myofibroblast transition in NHLF. In vivo, succinate treatment significantly increased collagen accumulation in the lung and enhanced weight loss in bleomycin-treated mice. Importantly, succinate-mediated elevation of fibrosis-associated markers was lost upon knockdown of SUCNR1 or inhibition of ERK activation in IPF patient-derived fibroblasts.

**Conclusion:**

Succinate exerted pro-fibrotic effects in vitro and in vivo. Thus, SUCNR1 antagonism may be a potential therapeutic target for the treatment of IPF.

## 1 Introduction

Idiopathic pulmonary fibrosis (IPF) is a chronic, progressive, and restrictive interstitial pulmonary disorder that is estimated to affect 14-63 per 100000 individuals and to have a median survival rate of 2-3 years if untreated (1, 2). IPF is known to progress with an increase in fibrotic tissue, which is presented as repetitive scarring of the lung tissue and might result in pulmonary hypertension. Furthermore, there is deposition of extracellular matrix (ECM), leading to obliteration of the normal alveolar architecture (3). Consequently, the capacity of the alveoli to perform gas exchange is drastically reduced and this can go hand in hand with additional consequences such as pulmonary hypertension (3, 4). While the exact etiology of IPF is unclear, occupational and external factors such as cigarette smoking, genetic predisposition, presence of viral infections and exposure to various forms of dust and fumes contribute to the increased risk of fibrosis (5). Currently, nintedanib and pirfenidone (approved by the FDA in 2014) are the only anti-fibrotic therapeutic agents specifically used to treat IPF (5). However, these drugs only reduce disease progression in IPF patients and neither improve lung function nor offer a cure (5).

More recently, the potential dysregulation of metabolic pathways in several cell types has become an area of great interest in fibrosis research (6, 7), as transcriptomic and metabolic profiling has revealed that the metabolism of carbohydrates, proteins and lipids are characteristically dysregulated in pulmonary fibrosis (7).

The tricarboxylic acid (TCA) or Krebs cycle lies at the center of several metabolic pathways including lipid metabolism, carbohydrate metabolism and amino acid metabolism. The TCA cycle has gained attention for its involvement in the progression of several diseases including cancer, autoimmune diseases, and inflammatory disorders (8). Some members of the pathway, including acetyl CoA, succinate, α-ketoglutarate, itaconate and fumarate play non-metabolic roles in intracellular signaling, paracrine activation of immune cells, and chromatin modification (8). Recent transcriptomic and metabolomics profiling of fibrotic lung tissue revealed that the TCA cycle and its members contribute to the progression of lung fibrosis (9–12). In line with this, α-ketoglutarate, a rate determining intermediate member in the Krebs cycle has also been affiliated with the pathogenesis of IPF (11). α -ketoglutarate levels are elevated in TGF-β -treated fibroblasts and are involved in pro-fibrotic processes such as stabilization of collagen and enhancing fibrotic phenotypes in myofibroblasts (11). Similarly, loss of cis-aconitate decarboxylase (which catalyzes the synthesis of itaconate from aconitate) and the consequent reduction in itaconate levels have been associated with worsening of lung fibrosis in the bleomycin model of lung fibrosis (10). Contribution of other TCA members such as succinate dehydrogenase (SDH), and fumarate within the context of pulmonary fibrosis have also been reported (13, 14). However, the role of other members in regulating the progression of IPF is still elusive with regards to the development of potential therapeutic opportunities.

Succinate, an intermediate member of the TCA cycle, can be found in the blood at concentrations ranging between 2-20 μM (15, 16). However, under pathological conditions such as myocardial infarction, Crohn’s disease, cancer, rheumatoid arthritis, liver fibrosis and, more recently, pulmonary fibrosis, the concentrations of succinate in blood and in tissues have been reported to be dramatically elevated (12, 17–21). A study by Wang et al. in 2021 revealed that succinate accumulates in fibrotic lungs of bleomycin-treated rats 28 days after bleomycin administration when compared to mice which received only PBS (12). Extracellular succinate exerts its effect on several cell types through the succinate receptor (SUCNR1), also known as the G-protein coupled receptor 91 (GPR91) (22). The activation of SUCNR1 on alveolar type II (ATII) cells has been recently shown to drive lung injury via a hypoxia-inducible factor 1α (HIF1α)-mediated proinflammatory pathway (23). Furthermore, it has also been revealed that succinate acts through numerous downstream signaling pathways including adenylate cyclase (AC) modulation, activation of mitogen activated pathway (MAP) kinases, and others (24).

In this study, we aim to clarify the role of succinate in IPF, both *in vitro* cellular effects of succinate on fibroblasts, and *in vivo* in the bleomycin model of pulmonary fibrosis.

## 2 Materials and Methods

### Reagents

Chemicals were obtained from Sigma (Vienna, Austria) unless specified otherwise. Vectashield/DAPI mounting medium as well as secondary antibodies and HRP/AP conjugated streptavidin were obtained from Vector Laboratories (Burlingam, CA, USA). Primary antibodies were purchased from Merck Millipore (Burlington, MA, USA), Cell Signaling (Cambridge, UK) Santa Cruz (Santa Cruz, CA, USA), Abcam (Cambridge, UK), Jackson ImmunoResearch (Baltimore Pike, PA, USA), Novus biologicals, (Colorado, USA) and Sigma. For detailed catalogue numbers, refer to supplementary table 3.

### Animals/Bleomycin model

10-12 weeks old female C57/Bl6 J mice were used for all animal experiments. All animal care complied with national and international guidelines (GZ2022-0.481.527,2024-0.754.000) and mice were purchased from Charles River. In brief, mice were anesthetized with isoflurane, and bleomycin or vehicle (saline solution) was instilled intratracheally with a Hamilton syringe at a dose of 1.5 U/kg. Body weight was measured daily. Sodium succinate (20 or 100 mg/kg) was injected intraperitoneally once daily starting at day 11 post bleomycin. Mice were subjected to lung function measurements on day 21 post bleomycin as previously described (25, 26). After euthanizing mice with an overdose of pentobarbital (150 mg/kg i.p.), the lung was perfused with PBS and a bronchoalveolar lavage was performed with an 18G cannula in the trachea, 1 ml of PBS containing 0.1 mM EDTA was instilled into the lung and collected. Lungs were weighed, right upper lobes were excised, fixed in Histofix and paraffin-embedded for histologic assessment, while for all other measurements, the remaining lung was snap frozen and subsequently pulverized in liquid nitrogen.

### Hydroxyproline assay

In brief, 10-15 mg of pulverized lung tissue were dissolved in 100-150 µl dH2O, an equal volume of 12N HCl was added and after incubation for 3 hours at 120°C, samples were centrifuged (10 000 x g, 3 min) and 10 µl of the supernatants were transferred to a 96 well plate. A hydroxyproline standard curve was generated by adding 0, 0.2, 0.4, 0.6, 0.8 and 1 µg in a series of wells. 100µL of Chloramine T was added to the samples and standard, and incubated at room temperature for 5 minutes. Afterwards, 100µL of 4-(Dimethylamino) benzaldehyde (DMAB) was added to each well and incubated at 60°C for 90 minutes. The absorbance was recorded at 560nm on a microplate reader. Hydroxyproline content per lung was then calculated using the following formula: (B/V*D) *total lung weight where B is the hydroxproline concentration as derived from the standard curve (µg), V is the sample volume in each well (µL) and D is the sample dilution factor.

### Succinate measurement in murine lung tissue

Succinate concentration in murine lung tissue was measured using the colorimetric Succinate Assay Kit-ab204718 (abcam). The assay was carried out in accordance to manufacturer’s protocol for succinate measurement from tissue samples.

### In situ hybridization/Immunofluorescence

#### Tissue

In situ hybridization (ISH) was performed using RNAscope kit for SUCNR1 according to the manufacturer’s protocol followed by immunofluorescence (IF) staining for respective markers as previously described (26, 27). IF staining of tissue sections was carried out as described previously (26, 27). Sections were incubated with TrueView autofluorescence quenching kit, followed by mounting with DAPI Vibrance (Vector Laboratories).

#### Cells

IF was performed with 30,000 IPF patient-derived fibroblasts in chamber well plates. After respective treatments with indicated compounds, cells fixed with 4% formaldehyde for 10 minutes at room temperature. Cells were washed thrice with PBS and then permeabilized in 0.1% triton X (in PBS) for 10mins at room temperature. Cells were again washed 3 times in PBS. Cells were then blocked for 1 hour in blocking buffer (10%goat serum, 1%BSA in PBS) and cells were incubated with primary antibody (Rabbit anti-aSMA 1:1000) prepared in incubation buffer (1%goat serum 1%BSA) overnight at 4 degrees. After washing the cells thrice In PBS, cells were then incubated with secondary antibody (Goat anti-rabbit Alexaflour 488 1:500) for 1 hour at room temperature.

After a final wash (3 times in PBS), cells were then mounted with vectashield mounting media containing DAPI. Cells were stored in 4 degrees until imaging. The images for all treatment groups from each experiment were recorded at the same time and using the same settings in a blinded fashion. Images were analysed using the Image J software.

### Collagen ELISA

Enzyme linked immunosorbent assay (ELISA) for the determination of the pro collagen 1 alpha 1 (n-terminal pro-peptide) was performed using the Duo set kit from bio-techne (Minneapolis, MN, USA) according to the manufacturer’s instructions. Supernatants from fibroblasts were used, standard curve preparation was done in the presence of medium and values were obtained by subtracting absorption at 540 nm from that at 450 nm on a microplate reader.

### Caspase3/7 activity measurement

Caspase3/7 activity measurement was performed using the Caspase-Glo® 3/7 3D Assay kit (Promega) according to the manufacturer’s instructions. After respective treatments, the cell culture medium was replaced by 100µl of fresh basal medium to which 100µl of Caspase-Glo® 3/7 3D reagent was added to each well. The contents of the plate were mixed for 30 seconds at 500rpm and incubated for 30 min at RT. The luminescence was detected using the CLARIOstar plus microplate reader (BMG Labtech).

### Cell culture

Fibroblasts isolated from 5 healthy donor lungs and 6 IPF/UIP (thereafter referred to as IPF)-patients (ethics approval 1417/2022 and 976/2010, for characteristics of patients/donors please see Supplementary Table 2, diagnosis assigned by transplant center) were isolated as previously described (28). Cells were kept in DMEM supplemented with 10% heat inactivated bovine serum and 1% Pen/Strep. IPF fibroblasts were serum starved for 24h, and treated for 72h treatment with indicated compounds. Afterwards, supernatants were collected and snap-frozen for ELISA, while cells were either lysed in TRIZOL or RIPA buffer.

Normal human lung fibroblasts from 5 healthy controls were isolated/treated as previously mentioned (28). Cells were kept in DMEM supplemented with 10% heat inactivated bovine serum and 1% Pen/Strep.

Fibroblast to myofibroblast transition in normal human lung fibroblasts: Cells were serum starved for 24h, and treated for 72h with or without 5ng/mL TGF-β, along with vehicle or varying concentrations of sodium succinate. Reverse transfection was performed 48h prior to starting the experiments in medium w/o antibiotics and serum using the Lipofectamine RNAiMAX kit (Thermo Fisher) according to the manufacturer’s instructions. siRNA select and RNAiMAX lipofectamine were diluted to a final concentration of 30 nM and 1:100 respectively, preincubated in OptiMEM for 20 minutes at RT and 60,000 cells were seeded. 10µM of the ERK inhibitor U0126 (Cell signaling) was administered 4h before treatment of IPF patient fibroblasts with sodium succinate on each day. For donor fibroblasts, cells were transfected 48 h before as described above 48 h and 2 h before 5ng/mL TGF-β treatment and then harvested for WB/ELISA 72h after addition of TGF-β.

In all experiments, each n represents a different IPF patient or healthy donor derived cell line. In the case of succinate receptor (SUCNR1) knockdown in fibroblasts, 5 independent replicates were performed in cells from 3 different donors/patients. In the ERK inhibitor experiments, 8 independent experimental replicates were performed in cells from 6 different IPF patients. Additionally, a detailed list of patient and donor cell lines are included in the supplementary data. All cells were routinely tested for mycoplasm contamination by microscopy (DAPI staining w/o antibiotics) when freshly thawed and intermittently via PCR (Venor GeM Mycoplasma Detection Kit (Bioproducts)).

### Western blot

Briefly, cells were lysed in RIPA buffer (Thermo Fisher Scientific), containing Halt protease and Phosphatase inhibitor cocktail (Thermo Fisher Scientific) and protein content was analyzed using the Pierce BCA-kit (Thermo Fisher Scientific) and 5 µg protein per sample were separated by SDS-PAGE on a 4-20% TRIS-glycine gradient gel (Thermo Fisher Scientific). For the siRNA knockdown experiments, cells were lysed in Trizol (Thermo Fisher Scientific) and protein was isolated according to the manufacturer’s instructions. Protein was then blotted using the iBlot dry transfer system (Thermo Fisher Scientific) on a polyvinylidene fluoride membrane; thereafter the membrane was blocked (5% non-fat dry milk) for 1 hour and incubated with primary antibodies overnight. Secondary HRP-conjugated antibodies followed by HRP-detection substrate (Bio-Rad) was used to visualize chemiluminescence by ChemiDoc Touch Imaging system (Bio-Rad). After stripping with respective buffer (Restore PLUS, Thermo Fisher Scientific), membranes were re-probed with antibodies for respective loading controls and processed as described above. Densitometric analysis of protein bands was performed using Imagelab Software (Bio-Rad).

Additionally, the list of antibodies and associated details including catalogue number, dilution, and validation method are included in the supplementary data (supplementary table 1).

### Real-Time Polymerase Chain Reaction (RT-PCR)

For relative quantification of mRNA, real-time PCR was performed using the CDX Connect Real-Time PCR detection system with CFX Manager Software 3.1 (Biorad). RNA isolation was done using Trizol (ThermoFisher Scientific) and DNA was removed with Ambion DNA-free DNA removal kit (ThermoFisher Scientific). RNA was reverse transcribed using the iScript cDNA synthesis kit (Biorad). Real-time PCR was performed using SsoAdvanced universal SYBR Green Supermix and PrimePCR SYBR Green Assay primers (Biorad) for human SUCNR1, GAPDH, and hPRT according to the manufacturer’s instructions.

### Microarray dataset

A publicly available dataset (GSE32537) from IPF/UIP patients (n=119) and controls (n=50) was used to compare SUCNR1 expression (29). Data was analyzed in RStudio (v.4.1.3) using Wilcoxon Signed Rank test, followed by Benjamini-Hochberg correction. Correlations were calculated using corrr package (30) and deconvolution was performed with Bisque (31).

### Data analysis

Statistical analysis was performed using Graph Pad Prism® 9 (GraphPad Software, Inc. CA, USA). For comparing two samples, Student’s t-test or Mann Whitney test was employed. For multiple comparisons one-way ANOVA followed by Holm-Sidak’s post hoc test for comparing all groups was used. Two-way ANOVA for repeated measurements with Holm-Sidak’s post hoc test was used for time courses. Significance was set at p< 0.05. Data are given as mean ± SD. Where applicable, normality was confirmed by using D’Agostino & Pearson testing.

## 3 Results

### 3.1 SUCNR1 is expressed in the human lung

**Figure 1:**
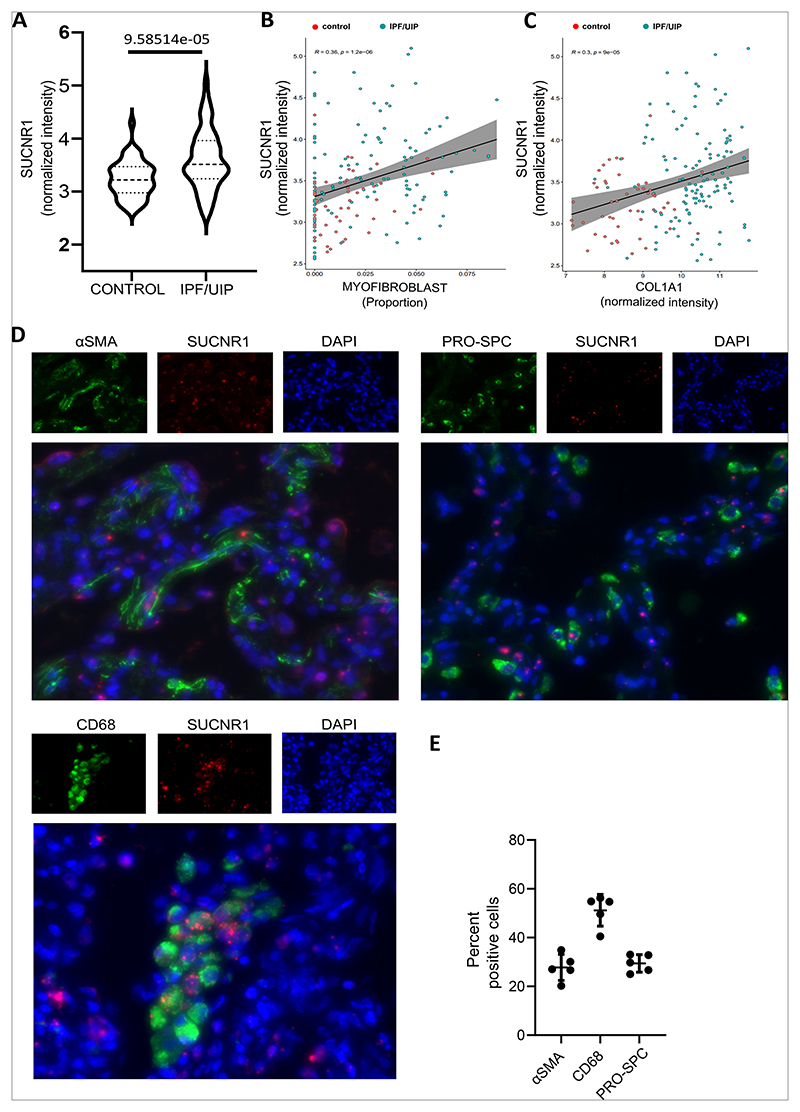
Succinate is expressed in the human lung. (A) Analysis of microarray datasets of lung tissue from healthy controls and lung tissue from patients diagnosed with IPF/UIP. Correlation between SUCNR1 gene expression and myofibroblasts (B) and COL1A1 (C) in lung tissues from healthy controls and diseased lung tissues derived from microarray dataset (GSE32537). (D) In situ hybridisation of human IPF lung tissue sections using a SUCNR1 probe, followed by immunofluorescence staining for alpha smooth muscle actin, prosurfactant protein C, and CD68. (E) Quantification of images in D to determine percentage of SUCNR1-positive cells.

In this study, we aimed to understand the role of the TCA cycle metabolite succinate and its receptor, SUCNR1, in the context of IPF. For this, we first aimed at determining if SUCNR1 is expressed in the healthy and fibrotic human lung. Analysis of publicly available gene expression datasets of lung tissue from IPF patients and healthy controls revealed that SUCNR1 is significantly upregulated in IPF patients when compared to healthy controls (Figure 1A). Interestingly, SUCNR1 expression also significantly correlated with the proportion of myofibroblasts present (as estimated by deconvolution) and COL1A1 expression levels (Figure 1B and C) (29, 31). To determine the expression of SUCNR1 in human lung tissue, we performed ISH of SUCNR1 coupled with immunofluorescence (IF) staining of different cellular markers, such as prosurfactant protein C (pro-SPC), alpha-smooth muscle actin (αSMA), and CD68. ISH coupled with IF of IPF lung tissue revealed that SUCNR1 is expressed in several cell types in the lung including cells that are positive for pro-SPC, αSMA, as well as CD68 (Figure 1D and E, Supplementary Figure 2G). In detail, we found that approximately 30% of both αSMA and pro-SPC positive cells were SUCNR1 positive, while this reached approximately 50% in macrophages (Figure 1E).

We also performed Western blot analysis of SUCNR1 in IPF lung tissue and healthy controls. While the receptor was expressed in the lung tissue of IPF patients and healthy controls, there was no observable difference (Supplementary figure 2A and B). In addition to this, we also aimed at determining the expression of SUCNR1 in fibroblasts as they are the major cell type that promote fibrotic processes during the progression of IPF. To this end, western blotting for SUCNR1 was performed with homogenates of normal human lung fibroblasts as well as IPF patient-derived fibroblasts. SUCNR1 was expressed in both normal human lung fibroblasts as well as in IPF patient-derived fibroblasts (Supplementary 2C and D). However, there was no observable difference in the expression of SUCNR1 in IPF patient fibroblasts in comparison to normal human lung fibroblasts. Additionally, we sought to assess SUCNR1 expression in murine lung tissue as a consequence of lung fibrosis in the bleomycin murine model of pulmonary fibrosis. Western blotting for SUCNR1 was performed with lung homogenates from mice which received either saline or bleomycin intratracheally (Supplementary 2E and F). In line with the SUCNR1 expression in human lung tissue homogenates, we observed that SUCNR1 expression was not altered in fibrotic murine lung tissue homogenates when compared to healthy controls (Supplementary 2E and F).

Taken together, these data show that SUCNR1 is expressed in the normal and IPF human lung. Moreover, it is expressed in several cell types in the human lung such as mesenchymal cells, alveolar epithelial cells and macrophages while it showed a positive correlation to myofibroblasts at the gene expression level in a publicly available dataset.

### 3.2 Succinate affects the expression of fibrosis-associated markers in IPF patient-derived fibroblasts

**Figure 2:**
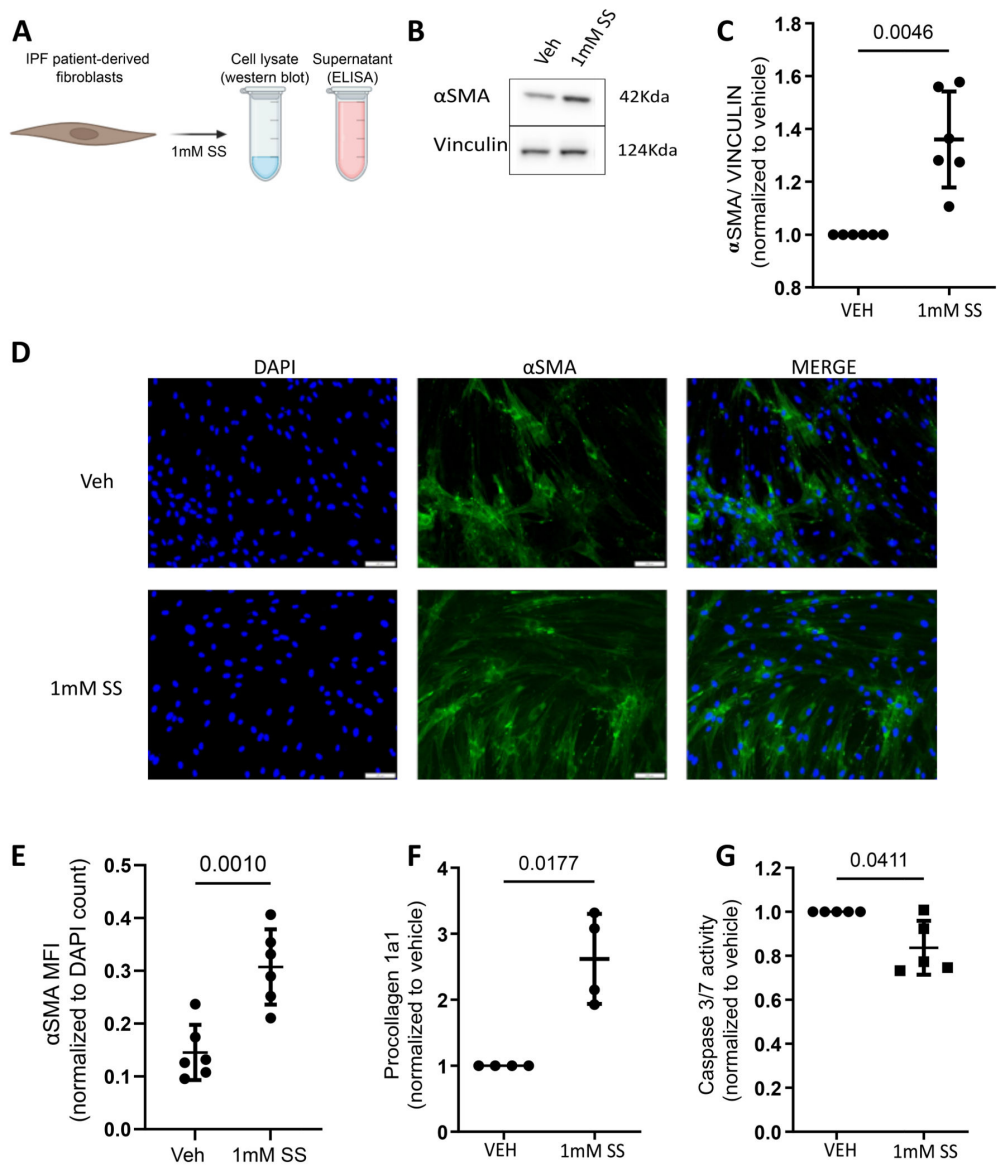
Succinate affects the expression of fibrosis-associated markers in IPF patient-derived fibroblasts. (A) Schematic representation of experimental setup to study the effect of succinate treatment of IPF patient-derived fibroblasts. (B) Western blot for αSMA with protein lysates from IPF patient-derived fibroblasts which were treated with 0 (veh) or 1mM sodium succinate (SS). Vinculin was used to control for loading. N=6. (C) Quantification of Western blot represented in B. (D) IF analysis of IPF patient-derived fibroblasts which were treated with 0 (veh) or 1mM SS. The samples were stained with an antibody against αSMA, and mounted with medium containing DAPI. N=6. (E) Quantification of IF images from (D). (F)ELISA for procollagen 1a1 with cell culture supernatants from IPF patient-derived fibroblasts treated with 0 (veh) or 1mM sodium succinate (SS). N=4. (G) Evaluation of caspase 3/7 activity of IPF patient-derived fibroblasts, treated with 0 or 1mM SS. Samples from each independent experiment were normalized to respective vehicle controls. statistical analysis was performed using paired t-tests.

Next, we wanted to assess the effect of SUCNR1 activation on the expression of fibrosis-associated markers in IPF patient-derived fibroblasts. To this end, we treated IPF patient-derived fibroblasts with sodium succinate, which yields succinate ions in solution, the ligand of SUCNR1 (Figure 2A). We then analyzed the expression of fibrosis-associated markers such as αSMA and type 1 collagen using Western blots, enzyme linked immunosorbent assay (ELISA), and IF staining respectively (Figure 2A). Western blot analysis of αSMA revealed that the treatment of IPF patient-derived fibroblasts resulted in a significantly elevated expression of αSMA relative to vehicle-treated control (Figure 2B and C). In line with the western blot analysis of αSMA, IF analysis also reveals that αSMA increases upon stimulation of IPF fibroblasts with 1mM SS (Figure 2D and E). Similarly, ELISA performed on the IPF patient derived fibroblast cell culture supernatant for procollagen 1a1, a precursor of the major protein constituent of type 1 collagen (32), revealed that sodium succinate led to an elevated expression of procollagen 1a1 (Figure 2F).

IPF fibroblasts have been characteristically associated with their increased resistance to apoptosis (33, 34). We measured caspase 3/7 activity in cells treated with and without 1mM sodium succinate. We observed that treatment of IPF patient-derived fibroblasts reduced their caspase 3/7 activity (Figure 2G). These data indicate that treatment of IPF patient-derived fibroblasts with succinate leads to an elevation of fibrosis-associated markers *in vitro*.

### 3.3 Succinate enhances TGF-β-induced fibroblast-to-myofibroblast transformation in normal human lung fibroblasts

**Figure 3:**
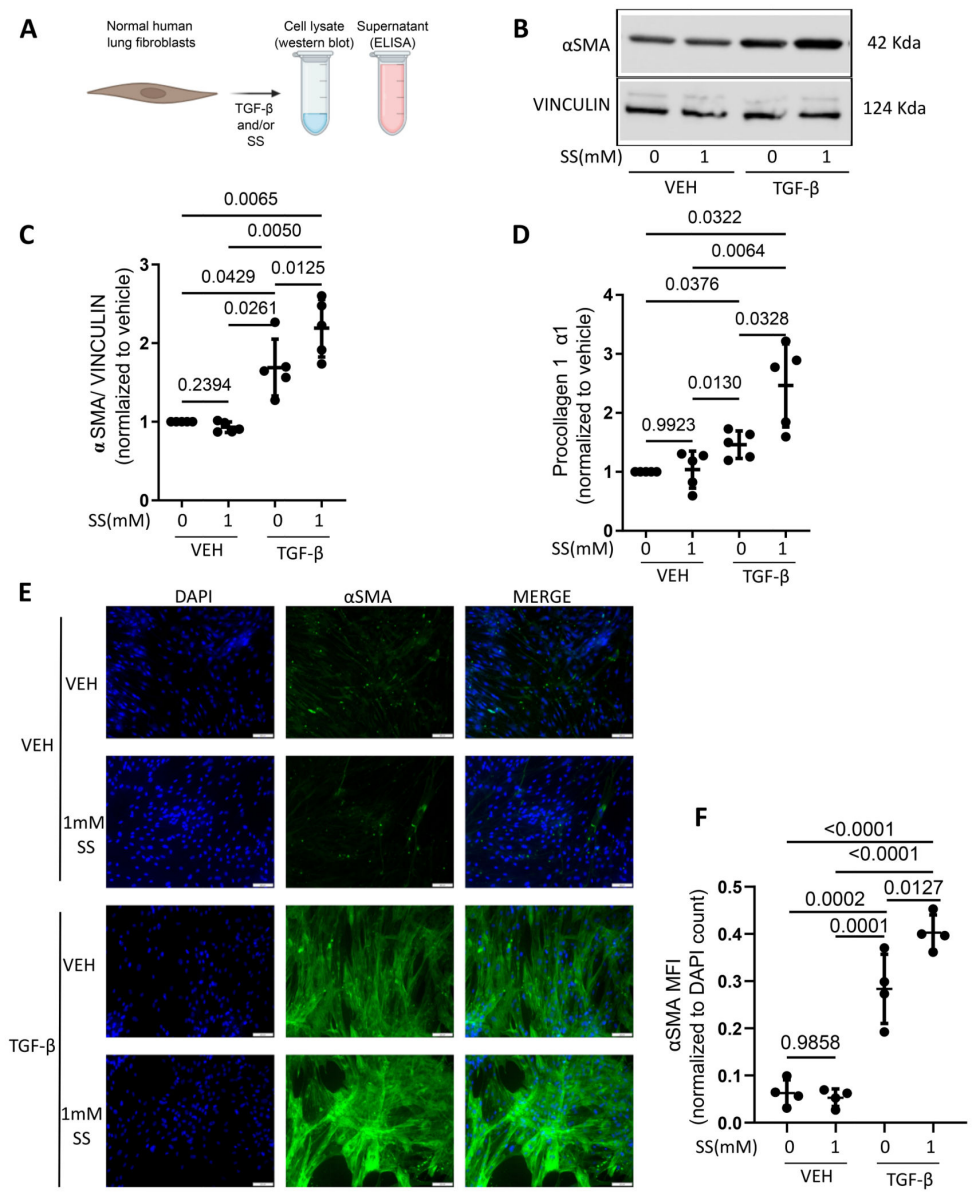
Succinate enhances TGF-β-induced fibroblast-to-myofibroblast transformation. (A) Schematic representation of experimental setup to study the effect of succinate treatment on TGF-β-induced fibroblast-myofibroblast transformation in normal human lung fibroblasts. (B) Western blot for αSMA with protein lysates from normal human lung fibroblasts treated with TGF-β and or sodium succinate (SS). Vinculin was used to control for loading. N=5. (C) Quantification of western blot shown in (B) Samples from each independent experiment were normalized to respective vehicle controls. (D) ELISA for procollagen 1a1 with cell culture supernatants from normal human lung fibroblasts treated with TGF-β and or sodium succinate (SS). (E) IF analysis of IPF patient-derived fibroblasts which were treated with 0 (veh) or 1mM SS. The samples were stained with an antibody against αSMA, and mounted with medium containing DAPI. N=4. (F) Quantification of IF images from (E). Statistical analysis was performed using One-way ANOVA for repeated measures, followed by Holm-Sidak test for multiple comparisons.

Fibroblast-to-myofibroblast transformation is closely related to the development and progression of IPF (35). As SUCNR1 expression is increased in IPF patient derived fibroblasts, we wanted to evaluate the effect on succinate on the fibroblast-to-myofibroblast transformation process. For this, normal human lung fibroblasts were treated with TGF-β or sodium succinate or a combination of both (Figure 3A). We then assessed αSMA levels by Western blot analysis and IF analysis, and procollagen 1a1 levels by ELISA (Figure 3A). Sodium succinate treatment alone did not induce fibroblast-to-myofibroblast transformation in these normal human lung fibroblasts as depicted by the unaffected αSMA and procollagen 1a1 levels (Figure 3B-F). As expected, TGF-β treatment of normal human lung fibroblasts led to a significant elevation of both αSMA and procollagen 1a1 (Figure 3B-F). Interestingly, treatment of normal human lung fibroblasts with a combination of TGF-β and sodium succinate further elevated the levels of αSMA and procollagen 1a1 (Figure 3B-F). These data indicate that while succinate does not affect fibroblast-to-myofibroblast transformation per se, it enhances fibroblast-to-myofibroblast transformation in the presence of pro-fibrotic mediators that are known to be present in pulmonary fibrosis.

### 3.4 Succinate affects collagen accumulation *in vivo*, in the bleomycin model of lung fibrosis

**Figure 4:**
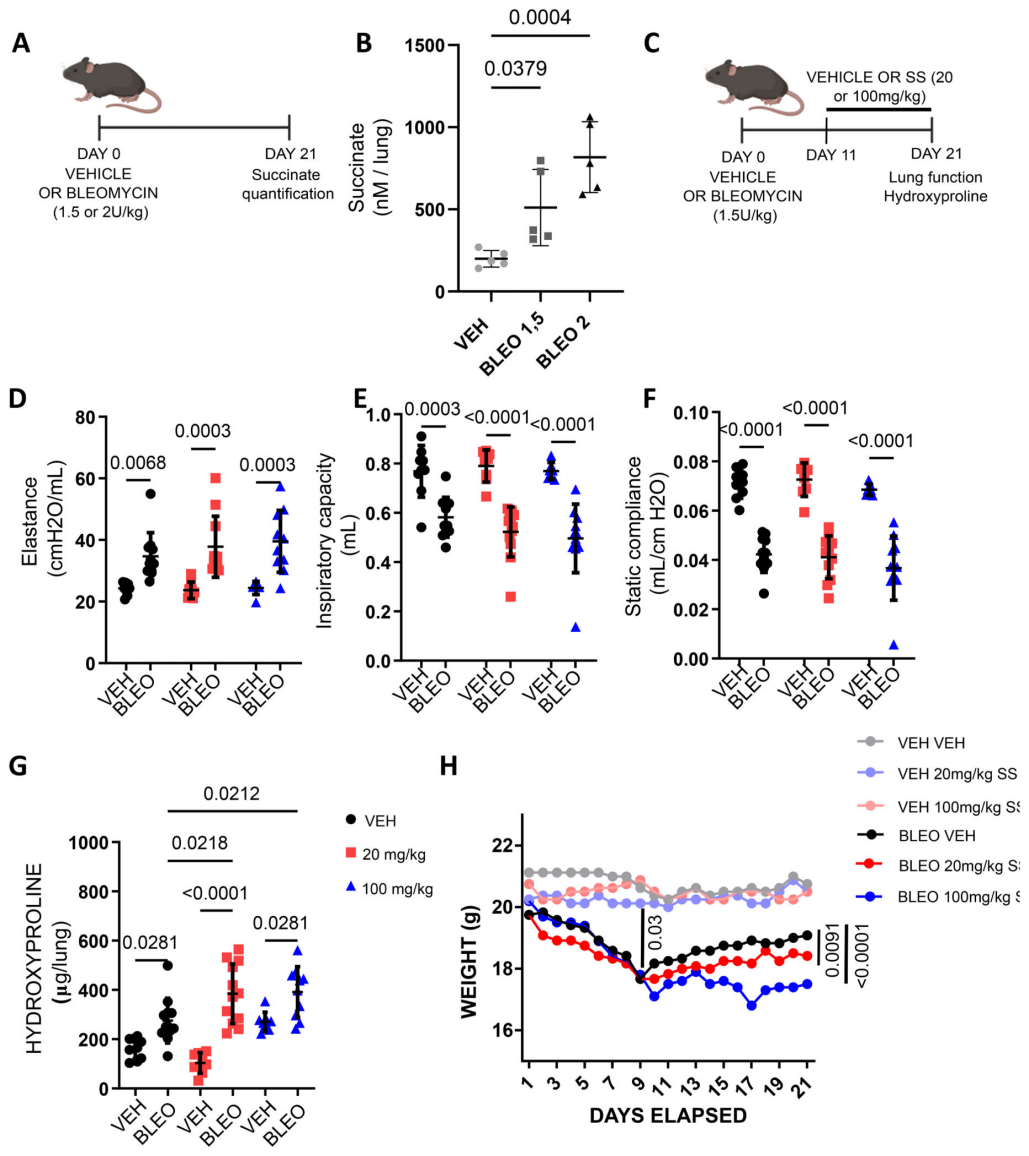
Succinate affects collagen accumulation in vivo. (A) Schematic representation of *in vivo* experimental setup to quantify succinate levels in lung tissue of C57BL6 mice treated with saline (vehicle (VEH)), 1.5 units/kg bleomycin (BLEO 1.5) or 2 units/kg bleomycin (BLEO 2). (B) Quantification of succinate levels in lung tissue homogenates of C57BL6 mice treated with saline (vehicle (VEH)), 1.5 units/kg bleomycin (BLEO 1.5) or 2 units/kg bleomycin (BLEO 2). (C) Schematic representation of *in vivo* bleomycin mouse model for validating the role of succinate in the progression of pulmonary fibrosis. Mice were instilled with bleomycin or saline intratracheally on day 0, followed by intraperitoneal administration of respective sodium succinate concentrations from day 11 onwards. Lung function parameters of interest were (D) elastance, (E) inspiratory capacity and (F) static compliance. (G) Hydroxyproline assay was performed on the lung homogenates of the respective groups of mice treated with bleomycin. (H) Weight of mice from respective treatment groups. VEH VEH-Vehicle + 0mg/kg sodium succinate, VEH 20mg/kg SS= Vehicle + 20mg/kg sodium succinate, VEH 100 mg/kg SS= Vehicle + 100mg/kg sodium succinate, BLEO VEH= Bleomycin + 0mg/kg sodium succinate, BLEO 20mg/kg SS= Bleomycin + 20mg/kg sodium succinate, BLEO 100mg/kg= Bleomycin + 100mg/kg sodium succinate. Statistical analyses were performed using One-way ANOVA for B, D, E, F, and G, followed by Holm-Sidak test for multiple comparisons and Two-way ANOVA followed by Tukeys’s test for multiple comparison for H.

Our *in vitro* data suggest that succinate exerts pro-fibrotic effects on IPF patient-derived fibroblasts. To further investigate the effect of succinate on the progression of fibrosis, we used the murine bleomycin model of pulmonary fibrosis. First, we wanted to assess if bleomycin treatment of mice led to an elevation of succinate levels in the lung. For this, we intratracheally administered either saline (VEH), 1.5 units/kg bleomycin (BLEO 1.5) or 2 units/kg bleomycin (BLEO 2) (Figure 4A). We then assessed the succinate levels in the lung homogenates 21 days post bleomycin administration to assess the succinate levels after establishment of fibrosis in the lung (Figure 4A) (36). Our data revealed that bleomycin treatment led to a significant, dose-dependent elevation of tissue succinate levels in mice when compared to mice which received only saline (Figure 4B).

We then aimed to understand the effect of succinate on the progression of pulmonary fibrosis in the bleomycin model. For this we intratracheally administered bleomycin or saline to mice, followed by treatment with 0 or 20 or 100mg/kg of sodium succinate (intraperitoneal injections) starting at day 11 until day 21 post bleomycin administration (Figure 4C). Sodium succinate injection was started at day 11 in order to study the fibrotic phase of the disease rather than the earlier, inflammatory phase (Figure 4C) (36). Succinate treatment was started on day 11 to avoid confounding effects on alveolar injury and inflammation that predominantly occur in the earlier phase of the model (days 1-10) and to focus on its effect on fibroblasts which are the major drivers of pulmonary fibrosis (36, 37).

In IPF patients, loss of lung function is associated with disease progression and mortality (38). In line with this, testing of lung function parameters is widely used to assess pathological changes associated with bleomycin-induced lung fibrosis in mice (25). Thus, we sought to assess the effect of succinate on lung function parameters in the bleomycin model of lung fibrosis. As expected, bleomycin treatment of mice considerably worsened several lung function parameters such as elastance, inspiratory capacity, and static compliance (Figure 4D-F). Treatment of bleomycin-instilled mice with sodium succinate tended to slightly worsen lung function parameters assessed, but did not reach significance (Figure 4D-F). However, assessment of hydroxyproline levels, which is considered the gold standard for collagen levels, revealed that sodium succinate treatment of bleomycin-treated mice led to a significant increase in the levels of collagen *in vivo* (Figure 4G) (39). Additionally, we found that treatment of bleomycin-treated mice with sodium succinate led to an exaggerated weight loss in these mice when compared to healthy controls (Figure 4H). These data indicate that succinate promotes the accumulation of collagen in bleomycin treated mice and thereby worsens pulmonary fibrosis in the bleomycin model.

### 3.5 Succinate induces ERK1/2 and p38 phosphorylation in IPF patient-derived fibroblasts

**Figure 5:**
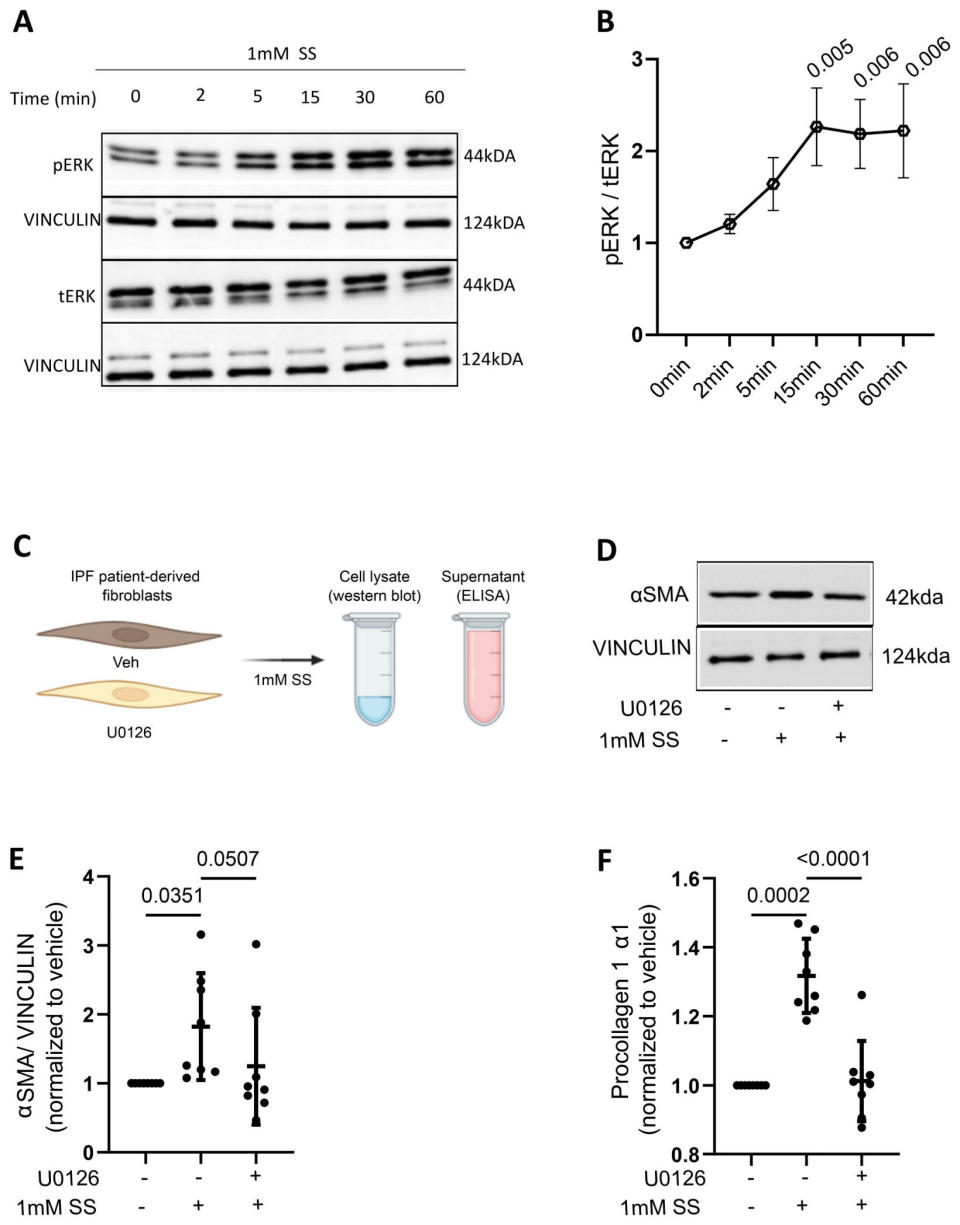
Succinate induces ERK phosphorylation in IPF patient-derived fibroblasts. (A) Western blot analysis of total ERK (tERK) and phosphorylated ERK (pERK) in protein lysates obtained from IPF patient-derived fibroblasts treated with 1mM sodium succinate (SS) over time points including 0, 2, 5, 15, 30 and 60 minutes. Vinculin was used as loading control. (B) Analysis and quantification of Western blot depicted in (A). (C) Schematic representation of experimental setup to study the effect of ERK inhibition on the succinate-mediated elevation of αSMA and type 1 collagen. (D) Western blot analysis for αSMA with cell lysates from IPF patient-derived fibroblasts treated with or without U0126, an ERK inhibitor, followed by 1mM SS. (E) Quantification of western blot depicted in (D). (F) ELISA for procollagen 1a1 with cell culture supernatants from cell culture supernatants of IPF patient-derived fibroblasts treated with or without U0126, an ERK inhibitor, followed by 1mM SS Samples from each independent experiment were normalized to respective vehicle controls. N=8. Statistical analysis was performed using One-way ANOVA for repeated measures, followed by Holm-Sidak test.

As we could observe pro-fibrotic effects of succinate both in vivo and in vitro, we wanted to examine the underlying mechanism. The ERK signalling cascade is activated during the progression of lung fibrosis (40). Additionally, ERK1/2 inhibition downregulates the deposition of collagen and alleviates lung fibrosis in the murine bleomycin model (40, 41). Moreover, succinate induces the activation of the ERK1/2 pathway in several cell types including hepatic stellate cells during non-alcoholic steatohepatitis-associated fibrosis (NASH), in keloid patient derived fibroblasts and in cardiomyocytes during cardiac hypertrophy (42–44). Thus, our goal was to understand if the ERK1/2 pathway is activated in IPF patient-derived fibroblasts upon succinate stimulation. For this, we stimulated IPF patient-derived fibroblasts with 1mM SS and assessed the ratio of phosphorylated ERK 1/2 (pERK) to total ERK 1/2 (tERK) at intervals of 2 minutes, 5 minutes, 15 minutes, 30 minutes, and 60 minutes using western blot analysis (Figure 5A and B). Upon stimulation of IPF patient-derived fibroblasts with 1mM SS, we observed an increase in pERK:tERK ratio with the increase in time, with the maximum phosphorylation at 15 minutes post-induction (Figure 5A and B). Thus, our data reveals that there is an increased phosphorylation of ERK1/2 upon stimulation by succinate in IPF patient-derived fibroblasts.

Finally, we wanted to determine if the succinate-mediated elevation of αSMA and type 1 collagen are a consequence of ERK activation. To address this, we treated IPF patient-derived fibroblasts with or without 10µM U0126 (1,4-diamino-2,3-dicyano-1,4-bis (2-aminophenylthio) butadiene), an inhibitor of ERK1/2 activation, after which the fibroblasts were treated with 1mM SS or control (Figure 5C). Western blot analysis revealed that cells treated with only succinate had a significantly elevated expression of αSMA, which was reduced to vehicle levels (but did not reach significance) when cells were subject to treatment with U0126 (Figure 5D and E). In line with this, treatment of IPF patient-derived fibroblasts with 1mM SS led to a significant elevation of type 1 collagen expression, which was abrogated upon treatment with U0126 (Figure 5F).

Apart from activation of the ERK signalling pathway, succinate can trigger the activation of other MAP kinases such as p38 which is involved in the regulation of fibroblast differentiation and ECM synthesis (45–47). Moreover, a recently published study has demonstrated that activation of SUCNR1 in pulmonary fibroblasts in the presence of TGF-β led to the phosphorylation of p38 (46). Thus, we assessed if the treatment of IPF fibroblasts with succinate led to the elevation of p38 phosphorylation. Interestingly and in line with the previous study, we could confirm that SUCNR1 stimulation of IPF-patient fibroblasts led to the activation of p38 (Supplementary Figure 3 A and B). Precisely, upon succinate treatment of the fibroblasts, we noted an increase in the phosphorylation of p38 with time, with the maximum phosphorylation at 15 minutes post-treatment (Supplementary figure 3A and B).

Thus, our data suggests that SUCNR1 activation in fibrotic lung fibroblasts regulates expression of fibrosis associated markers through the activation of MAP kinases.

### 3.6 SUCNR1 knockdown in IPF and normal lung fibroblasts reduces αSMA and type 1 collagen expression

**Figure 6:**
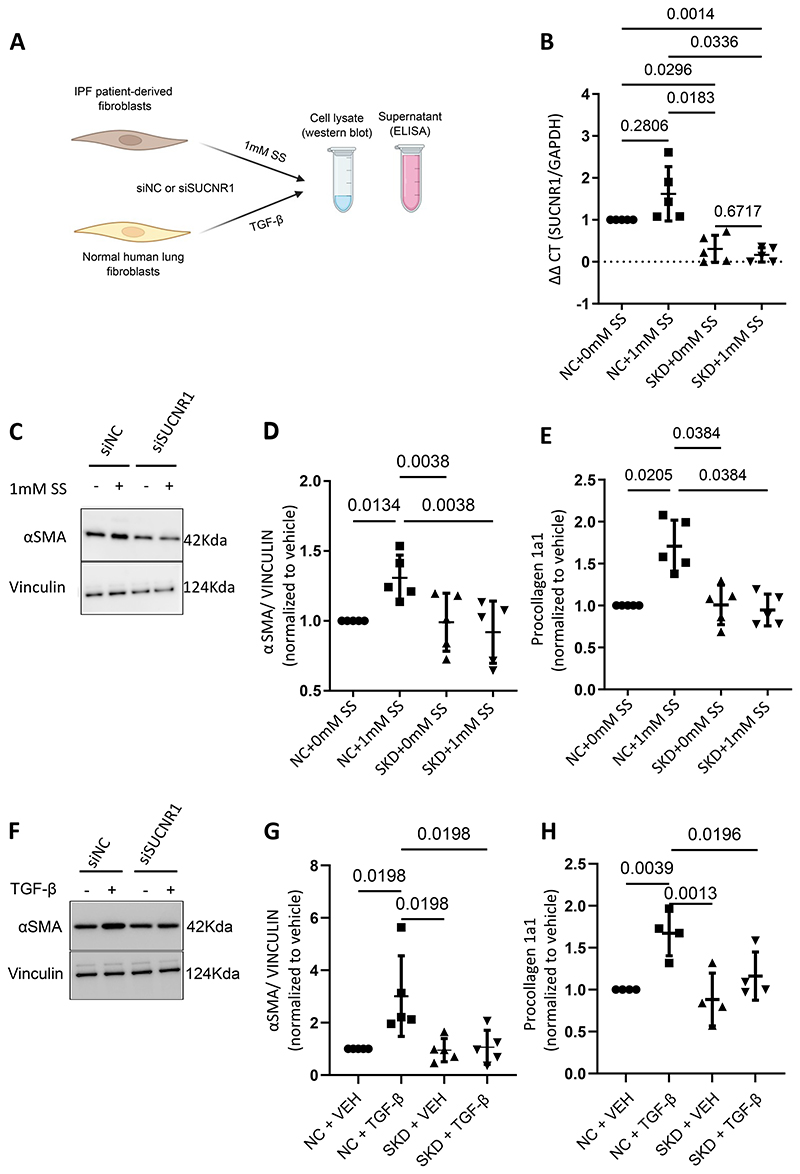
Knockdown of SUCNR1 in IPF patient-derived fibroblasts and normal human lung fibroblasts. (A) Schematic representation of experimental setup to assess the effect of siRNA-mediated SUCNR1 knockdown on succinate mediated elevation of fibrosis-associated markers in IPF patient fibroblasts and on TGF- -mediated activation of normal human lung fibroblasts (B) RT-PCR of IPF patient-derived fibroblasts transfected with either negative control (NC) or SUCNR1 siRNA (siRNA-mediated knockdown (SKD)), which were treated with 0 or 1mM sodium succinate (SS). N=5. (C) Western blot for αSMA with protein lysates from IPF patient-derived fibroblasts which were transfected with either control (NC) or SUCNR1 SiRNA, and treated with 0 or 1mM sodium succinate (SS). (D) Quantification of Western blot shown in (C). (E) ELISA for procollagen 1a1 from cell culture supernatants of IPF patient-derived fibroblasts which were transfected with either control (NC) or SUCNR1 SiRNA, and treated with 0 or 1mM sodium succinate (SS). (F) Western blot for αSMA with protein lysates from normal human lung fibroblasts which were transfected with either control (NC) or SUCNR1 SiRNA, and treated with or without TGF-β. (G) Quantification of western blot shown in figure F. (H) ELISA for procollagen 1a1 from cell culture supernatants of normal human lung fibroblasts which were transfected with either control (NC) or SUCNR1 SiRNA, and treated with or without TGF-β. N=5. Samples from each independent experiment were normalized to respective vehicle controls. Statistical analysis was performed using One-way ANOVA for repeated measures, followed by Holm-Sidak test for multiple comparisons.

Finally, in IPF patient-derived fibroblasts, we sought to determine if the elevation of fibrosis-associated markers such as αSMA and type 1 collagen upon treatment with sodium succinate was a receptor-mediated response. To address this, we performed siRNA-mediated knockdown of SUCNR1 *in vitro* (Figure 6A). SUCNR1 knockdown was validated using qPCR, which confirmed the loss of SUCNR1 gene expression (Figure 6B). Cells with or without the SUCNR1 knockdown were then treated with sodium succinate (Figure 6A). Western blot analysis of αSMA and ELISA for procollagen 1a1 revealed that the elevation of αSMA and type 1 collagen expression, which occurred in response to succinate treatment, was lost upon the knockdown of SUCNR1 (Figure 6C, D and E). These data suggest that the elevation of αSMA and type 1 collagen in response to sodium succinate treatment is a SUCNR1-mediated effect.

In addition, we investigated whether SUCNR1 knockdown affected TGF-β-mediated elevation of αSMA and type 1 collagen. We performed siRNA-mediated knockdown of SUCNR1 in normal human lung fibroblasts (Figure 6A). Surprisingly, even in the absence of exogenous succinate, knockdown of SUCNR1 in normal human lung fibroblasts led to the suppression of TGF-β-induced elevation of αSMA and type 1 collagen compared to negative control (Figure 6F, G and H). This is in contrast to untreated IPF- and normal human lung fibroblasts, where SUCNR1 -KD had no effect on αSMA and collagen 1a1 in the absence of TGF beta and SS. Taken together, our data so far indicate that succinate exerts its effects via the succinate receptor on IPF patient-derived fibroblasts, thereby promoting the expression of fibrosis-associated markers *in vitro* and knockdown of the receptor prevents TGF-beta induced myofibroblast differentiation in normal human lung fibroblasts.

## 4 Discussion

In the current study, we have explored the role of the succinate receptor, SUCNR1 within the context of IPF. Analysis of gene expression datasets revealed an upregulation of SUCNR1 levels in lung tissue of IPF patients with a positive correlation to myofibroblasts. We identified that several cell types in the lung express SUCNR1.Moreover, our *in vitro* data revealed that succinate via SUCNR1 led to an increase in the expression of fibrosis-associated markers such as αSMA and collagen, which was attenuated upon knockdown of SUCNR1. Additionally, our study also demonstrates that the activation of MAP kinases such as ERK are essential for the succinate/SUCNR1-mediated profibrotic effects. Our *in vivo* data showed that treatment of mice with sodium succinate led to an increase in the expression of collagen and an exaggerated weight loss in bleomycin-treated mice when compared to healthy controls. Thus, both *in vitro* as well as *in vivo* data suggest that succinate promotes the expression of fibrosis/associated markers and thereby exacerbates pulmonary fibrosis. Our study validates the involvement of the succinate receptor and its sensing of succinate as a potential driver of fibrotic phenotypes *in vitro* and *in vivo*. Other studies in the recent decade have affiliated the succinate-SUCNR1 axis to the pathogenesis of other fibrotic entities such as intestinal fibrosis and hepatic fibrosis (18, 42, 48). In concert with these studies, our study corroborates the role of succinate in promoting fibrotic pathogenesis.

Publicly available gene expression datasets revealed a significant upregulation in SUCNR1 expression in IPF/UIP patient lung tissue when compared to healthy controls. Interestingly, the expression levels of SUCNR1 increased with the proportion of myofibroblasts (as inferred by deconvolution performed on a publicly available microarray dataset (GSE32537) from IPF/UIP patients (n=119) and controls (n=50) (29, 31)) and COL1A1 gene expression, which further highlighted the potential role of the succinate/SUCNR1 axis in the pathogenesis of lung fibrosis. Moreover, we confirmed the expression of SUCNR1 on the protein level in human lung tissue samples by Western blotting. In further *in vitro* experiments we used IPF patient-derived fibroblasts to examine the contribution of the succinate-SUCNR1 axis towards the expression of important markers of pulmonary fibrosis such as αSMA and collagen. While we identified several cell types in the lung, which express the succinate receptor SUCNR1, we were interested in fibroblasts, which predominantly influence the production of ECM and therefore, the progression and functional outcomes in IPF. Indeed, we observed that stimulation of IPF patient-derived fibroblasts with succinate led to a significant elevation of the expression of fibrosis-associated markers such as αSMA and collagen. Additionally, we observed that succinate enhanced TGF-β-mediated activation of normal human lung fibroblasts. Finally, we were also able to prove the involvement of the receptor using the siRNA-mediated approach to knockdown SUCNR1. These findings are in line with several studies, which show that the cellular expression of SUCNR1 is elevated in several diseases such as Chron’s disease (CD), intestinal fibrosis and NASH wherein tissue fibrosis is one of the most severe complications (18, 42, 48). For instance, elevated succinate levels and elevated expression of SUCNR1 have been observed in intestinal resections from CD patients (18). Moreover, treatment of fibroblasts isolated from intestinal resections of CD patients led to an elevation of fibrosis-associated markers such as αSMA and collagen (18). Additionally, data from a study on NASH revealed that exposure to succinate elevated the expression of SUCNR1 in hepatic stellate cells (42). Consequently, there was a succinate-SUCNR1-mediated increase in the elevation of αSMA and collagen (42). Interestingly, when SUCNR1 knockdown was performed before TGF-beta stimulation of healthy human lung fibroblasts, the increase of αSMA and collagen was prevented. This hints strongly towards a role of the succinate/SUCNR1 axis in myofibroblast differentiation, very likely via TGF-beta induced increased succinate levels and subsequent receptor activation. However, the fact that succinate alone had no significant effects on healthy human lung fibroblasts shows that a co-stimulus is necessary.

*In vivo*, we observed that the levels of hydroxyproline were significantly elevated in mice treated with either a low or a high dose of sodium succinate.. In fact, the potential role of succinate and other members of the TCA cycle in influencing the biosynthesis of collagen and other ECM components would call for further interesting investigations. For example, α-ketoglutarate, a metabolite whose levels are elevated in IPF fibroblasts, enhances collagen stabilization as it is involved in the hydroxylation of proline residues (11). Moreover, α-ketoglutarate is known to epigenetically influence the expression of anti-apoptotic genes in IPF fibroblasts (49).

SUCNR1 is a seven-transmembrane receptor that couples to a trimeric G-protein which, upon stimulation, dissociates into its α subunit and a βγ dimer (50). Depending on the cellular context, these subunits, stimulate several downstream signalling cascades which impact cellular processes such as migration, proliferation, and modulation of protein activity among several other reported functions (50). Succinate concentrations in the circulation do not necessarily reflect the concentrations at the tissue or at the cellular level. Indeed, tissue succinate concentrations close to the mM range have been previously estimated (51–53). For instance, in obese patients, pulmonary succinate levels have been known to increase to concentrations as high as 0.4mM in the lung tissue while maximum serum succinate levels are as low as 60 µM (51). Moreover, this elevation of tissue succinate levels in obese patients are associated with the development of pulmonary complications such as acute respiratory distress syndrome (51). In addition to this, succinate which signals via SUCNR1, has an EC50 in the micromolar range (54). Hence, using succinate concentrations in the high micromolar range up to 1mM is expected to result in full receptor activation. It is a drawback of *in vitro* assays where high concentrations of stimuli are often required to induce distinct cellular responses as compared to plasma peak concentrations (*in vitro-in vivo scaling*) (55). In our study, treatment of IPF patient-derived fibroblasts with 1mM sodium succinate led to the activation of the MAP kinase signalling pathways such as the ERK signalling pathway as well as the p38 signalling pathway. Additionally, inhibition of the ERK signalling cascade led to the downregulation of succinate-mediated elevation of fibrosis-associated markers such as αSMA and type 1 collagen. In line with this, previous studies have revealed that the activation of the ERK pathway during pulmonary fibrosis enhances fibroproliferation, myofibroblast differentiation and production of extracellular matrix proteins (40). Moreover, data from the bleomycin model of lung fibrosis revealed that the pharmacological inhibition of the ERK signalling cascade ameliorated pulmonary fibrosis (41). The observations from our current study fits with the aforementioned studies on fibroblasts as well as the murine bleomycin mouse model which corroborates the significance of the ERK pathway in the progression of lung fibrosis as a potential target (40, 41).

In support of our *in vitro* data, which indicate that succinate exaggerates fibroblast activities, we also found elevated levels of hydroxyproline in the lung homogenates of bleomycin mice which were additionally treated with sodium succinate as compared to mice treated with bleomycin only. Similarly, weight loss after bleomycin treatment was more pronounced in succinate-treated mice, which clearly demonstrates that succinate treatment exaggerates the bleomycin-induced alterations in this model. However, we did not observe a significant worsening – except a slight tendency - of lung function in succinate-treated mice as compared to bleomycin alone. Noteworthy, bleomycin treatment led to a severe loss of lung function on its own, which might not be sensitive to further pathogenic stimuli. Thus, succinate treatment may not have further aggravated this effect on lung function, especially considering the already increased levels of succinate with bleomycin only.

A recent study by He and colleagues further corroborate the role of the succinate/SUCNR1 axis in promoting lung fibrosis (56). In their study, the authors demonstrate the altered serum levels of several TCA cycle metabolites including succinate in patients with IPF when compared to healthy controls (56). In line with our findings, they reveal that succinate via SUCNR1 enhances the expression of fibrosis-associated markers in vitro (in fibroblasts stimulated with TGF-beta) and enhanced fibrosis in vivo (in the bleomycin murine model) (56). In our study, we are able to further demonstrate the pro-fibrotic effects of succinate directly in fibroblasts derived from IPF patients which in line with He and colleagues is dependent on the SUCNR1 (56). While He and colleagues supplemented succinate through the entire course of the bleomycin model, this also includes the predominantly inflammatory phase post-bleomycin administration (56). In contrast to He and colleagues, our study demonstrates the effect of succinate supplementation during the fibrotic phase of the disease, thereby providing further evidence for a true pro-fibrotic effect (36). Thus, both the studies clarify the role of the succinate/SUCNR1 axis in enhancing pulmonary fibrosis.

Currently, the study suffers from a few limitations. It may be of interest to understand the effect of succinate supplemented the during earlier stages (only days 0-11) of the bleomycin model which is predominantly an inflammatory phase where the airway epithelium undergoes disruption and is followed by the recruitment and proliferation of fibroblasts (57, 58). This is in line with a recent study which highlighted the pro-inflammatory role of succinate in ATII cells during inflammatory lung injury (23). Additionally, other cell types including alveolar macrophages play an important role in the progression of pulmonary fibrosis (59). A study unveiled a profound effect of succinate on the polarization and function of macrophages (60). This presents the need to better understand the role of macrophages and ATII cells with regards to the succinate/SUCNR1 axis in pulmonary fibrosis. However, our study was focused on the fibroproliferative phase and thus did not explore succinate administration at earlier time points that would likely predominantly reflect effects on inflammation (36). Another limitation is the use of only female mice in our study. Further studies should be conducted to validate the pro-fibrotic effect of succinate in male mice. Furthermore, while we clearly show in vitro that succinate enhances collagen synthesis in fibroblasts via SUCNR1 and this correlates well with hydroxyproline levels in the bleomycin model, further experiments (such as for example fibroblast specific knockdown of SUCNR1 in mice) would be required to unequivocally prove that the same mechanism is present *in vivo*.

In conclusion, our study unravels the role of the succinate receptor in the pathogenesis of pulmonary fibrosis. Our *in vitro* data show that succinate exerts its effect on fibroblasts through the succinate receptor and leads to the enhanced expression of fibrosis-associated markers such as collagen and αSMA. In the bleomycin model of pulmonary fibrosis, we observed that sodium succinate indeed plays a role in enhancing the accumulation of collagen. Thus, our study highlights the role of the succinate-SUCNR1 axis in pulmonary fibrosis. This calls for further investigation to unravel the potential of SUCNR1 as a therapeutic target for pulmonary fibrosis.

## Supplementary Material

Supplementary Material

## Figures and Tables

**Figure:**
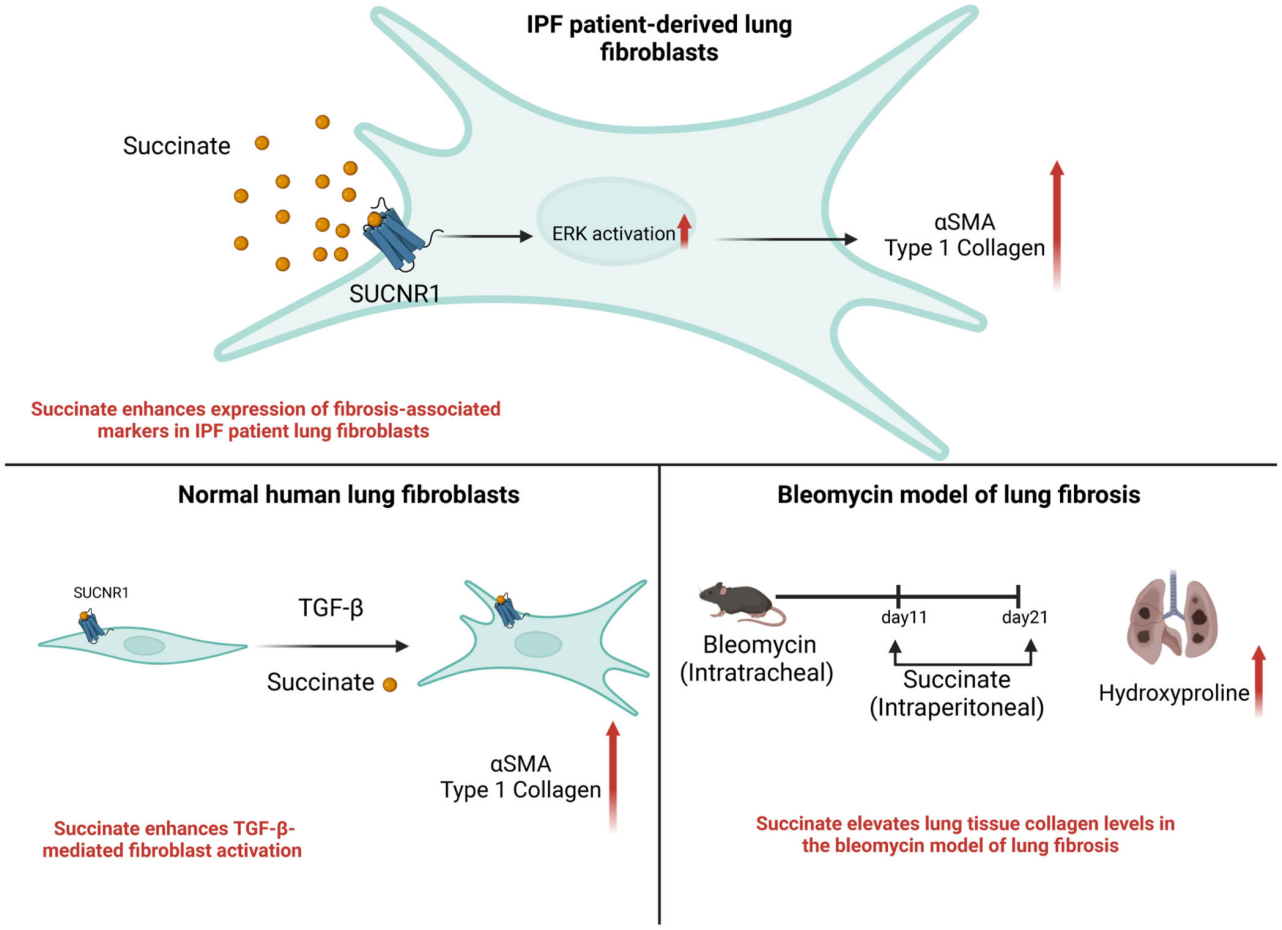


## New and noteworthy

This paper highlights the role of the succinate/SUCNR1 axis in pulmonary fibrosis. Receptor activation leads to pro-fibrotic changes in IPF-patient derived fibroblasts. This finding could also be replicated in a mouse model of pulmonary fibrosis.
