# Supplementary material for: Succinate aggravates pulmonary fibrosis through the succinate/SUCNR1 axis
Source: Am J Physiol Lung Cell Mol Physiol. Author manuscript; Available in PMC 2025 Jul 28. (PMC7617945; doi:10.1152/ajplung.00286.2024)
Supplement: Supplementary Material [file EMS206268-supplement-_Supplementary_Material.pdf]

Succinate aggravates pulmonary fibrosis through the succinate/SUCNR1 axis.

Supplementary data

Name of authors: Rishi Rajesh[1,6], Agnes Anna Mooslechner[1,6], Hannah Schweighofer[1,6], Svetlana Pahernik[1,6], Ilse Lanz[1,6], Reham Atallah[1], Wolfgang Platzer[1], Clemens Aigner[2], Alberto Benazzo[2], Stefano Angiari[3], Leigh Marsh[4], Grazyna Kwapiszewska[4,5], Akos Heinemann[1], and Thomas Bärnthaler[1,6*].

Affiliations: 1- Otto Loewi Research Center, Division of Pharmacology, Lung Research Cluster, Medical University of Graz, Austria. 2- Department of Thoracic Surgery, Medical University of Vienna, Vienna, Austria.3- Otto Loewi Research Center, Division of Immunology, Medical University of Graz, Austria. 4- Otto Loewi Research Center, Lung research Cluster, Graz, Austria. 5- Institute for Lung Health, Cardiopulmonary Institute, Member of German Lung Center (DZL), Giessen, Germany. 6- Research Unit Molecular Pharmacology in Pulmonary Disease, Medical University of Graz, Austria

*Corresponding Author:

Name: Thomas Bärnthaler, MD, PhD

Email address: thomas.baernthaler@medunigraz.at

Supplementary table 1: Details pertaining to the antibodies used in the study including manufacturer, dilution used, catalogue number, and validation of the antibody (method used, result of validation and resepctive references).

Antibody	Manufacturer	Dilution	Catalog number	Validation in	Method of validation	Result	Reference
Rabbit anti-aSMA	Abcam	1/5000	ab5694	Human umblical vein endothelial cells (HUVECs)	Western blot	aSMA Absent in HUVECs	1
anti-proSPC	Millipore	1/1000	AB3785	Human lung tissue and platelets	Western blot	proSPC absent in platelets	supplementary figure 1
Mouse anti-CD68	Abcam	1/500	ab955	Cells from bone marrow depelted of macrophages via clodronate liposome treatment	Western blot	Loss of CD68 protein expression	2
Rabbit anti-SUCNR1	Novus biologicals	1/5000	NBP-00861	Human umblical vein endothelial cells (HUVECs)	SUCNR1 knockdown followed by western blot and flow cytometry	Reduction in SUCNR1 upon knockdown	3
Rabbit anti-pERK	Cell Signaling	1/5000	4370S	Human melanoma cell line A375	ERK shRNA treatment followed by western blot	Loss of pERK upon knockdown	4
Rabbit anti-ERK	Cell Signaling	1/5000	4695S	Human melanoma cell line A375	ERK shRNA treatment followed by western blot	Loss of total ERK upon knockdown	4
Rabbit anti-Vinculin	Abcam	1/10000	EPR8185	Human mesenchymal stem cells	siRNA knockdown followed by western blot	Reduction in vinculin exprssion upon knockdown	5
Rabbit anti-GAPDH	Cell Signaling	1/10000	5174S	Squamous cell carcinoma cells	Western blot of cell nuclear fraction	GAPDH absent in nuclear fraction	6
Mouse anti-B-actin	Cell Signaling	1/10000	3700S	human osteosarcoma cell line U2OS	IP-Western blot.	Presence of B-actin in total lysate but not in IP fraction	7

Supplementary table 2: Characteristics of donor and IPF patients including age, sex, mean pulmonary arterial pressure(mPAP), (Forced) vital capacity (FVC), and New York Heart association (NYHA) functional classification.

Status	Age	Sex	mPAP [mmHg]	(F)VC (l)	NYHA Class
Donor	49	Female			
Donor	58	Female			
Donor	25	Female			
Donor	45	Female			
Donor	54	Female			
Patient	47	Male	62	2,12	III-IV
Patient	67	Male	65	1,6	IV
Patient	58	Male	68	2	III
Patient	51	Female	36	1,66	IV
Patient	52	Male	29	1,83	III
Patient	48	Male	38	1,98	II-III

Supplementary table 3: List of reagents utilized in this study with details including manufacturer and catalogue number.

S.No.	REAGENT	MANUFACTURER	CATALOGUE NUMBER
1	Vectashield mounting medium with DAPI	Vector laboratories	H-1200-10
2	Bleomycin	Baxter	84.882
3	L-hydroxyproline	Roth	3893.1
4	Chloramine T trihydrate	Szabo-Scandic	SACSC-211067
5	Dimethylamino benzaldehyde (DMAB)	Roth	X867.1
6	Succinate assay kit	Abcam	ab204718
7	RNAscope LS 2.5 Probe Hs SUCNR1	Biotechne	437728
8	RNAscope 2.5HD red in situ hybridization kit	Biotechne	322360
9	Vectashield Vibrance mounting medium with DAPI	Vector laboratories	H-1800
10	Bovine serum albumin	Sigma Aldrich	a7906
11	Dulbeco's Phosphate buffered saline	Thermo Fisher Scientific	14190169
12	Human Procollagen 1 alpha 1 ELISA	Bio-techne	DY6220-05
13	Caspase 3/7-Glo 3D Assay kit	Promega	G8091
14	Dulbeco's modified eagle's medium	Thermo Fisher Scientific	41965-062
15	TRIZOL	Thermo Fisher Scientific	15596-026
16	RIPA Lysis extract buffer	Thermo Fisher Scientific	89901
17	Penicilin/streptomycin	PAN-Biotech	P06-07100
18	Recombinant human TGF-β protein	Bio-techne	240-B-002
19	Lipofectamine RNAiMAX reagent	Thermo Fisher Scientific	13778-075
20	silencing RNA negative control	Thermo Fisher Scientific	4390843
21	silencing RNA for SUCNR1	Thermo Fisher Scientific	4392420
22	Opti-MEM Reduced serum media	Thermo Fisher Scientific	31985070
23	U0126	Cell Signaling	#9903
24	Venor GeM Mycoplasma Detection Kit	Bioproducts	MP0025
25	Pierce BCA protein assay kit	Life technologies	23227

Supplementary figure 1

A

LANE	SAMPLE
1	Human Platelets
2	Human Platelets
3	Human Platelets
4	Human lung tissue
5	Human lung tissue
6	Human IPF lung tissue
7	Ladder

B

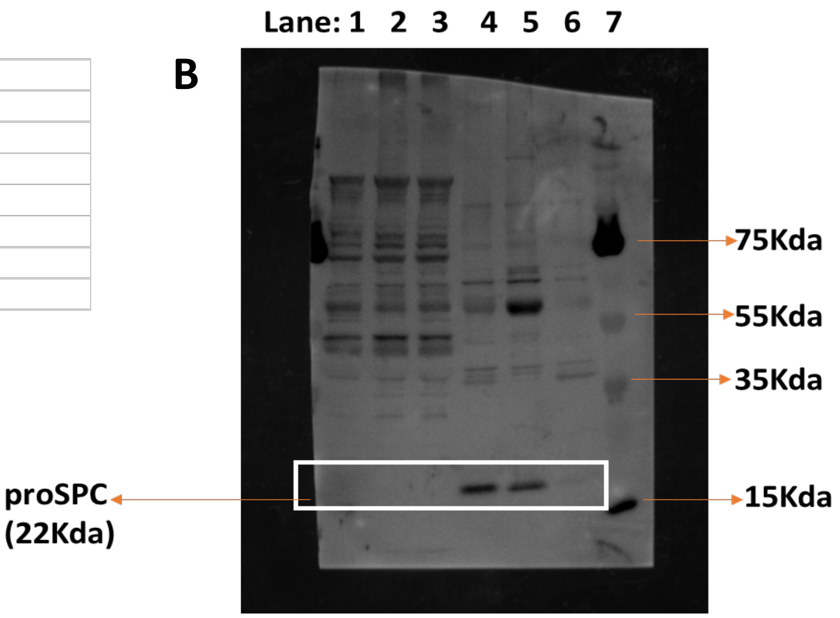

Supplementary figure 1: Validation of proSPC antibody by western blot.

(A) Lane description for western blot depicted in (B).

(B) Western blot for proSPC in protein lysates obtained from human platelets (lanes 1-3), human lung tissue (lane 4-5) or human IPF lung tissues (lane 6).

Supplementary figure 2

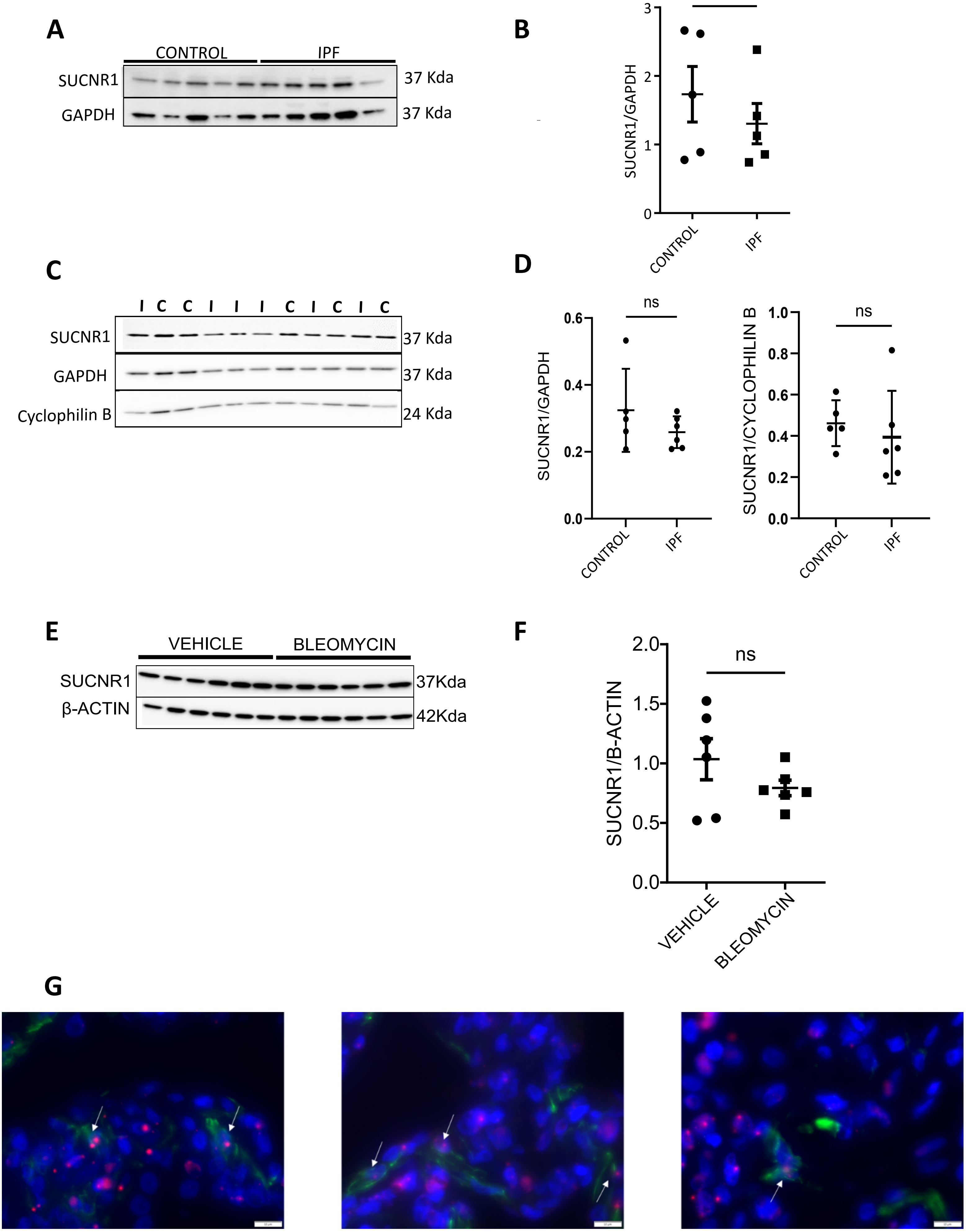

Supplementary figure 2: SUCNR1 is expressed in human and mouse lungs.

(A) Western blot for SUCNR1 with protein lysates from normal human lung tissue (control) and IPF patient lung tissue. GAPDH was used as loading control. N=6. (B) Quantification of western blot in (A).
(C) Western blot for SUCNR1 with protein lysates from normal human lung fibroblasts (control) and IPF patient-derived fibroblasts. GAPDH and Cyclophilin B were used to control for loading. N=5 (control), N=6 (IPF). C= control, I=IPF (D) Quantification of western blot in (C)
(E) Western blot for SUCNR1 with protein lysates from lung tissue of PBS (vehicle) or bleomycin treated mice. N=6. (F) Quantification of western blot in (E). (G) In situ hybridisation of human IPF lung tissue sections using a SUCNR1 probe (red), followed by immunofluorescence staining for alpha smooth muscle actin (green), and DAPI (blue).
Statistical analysis were performed using unpaired t-test.

Supplementary figure 3

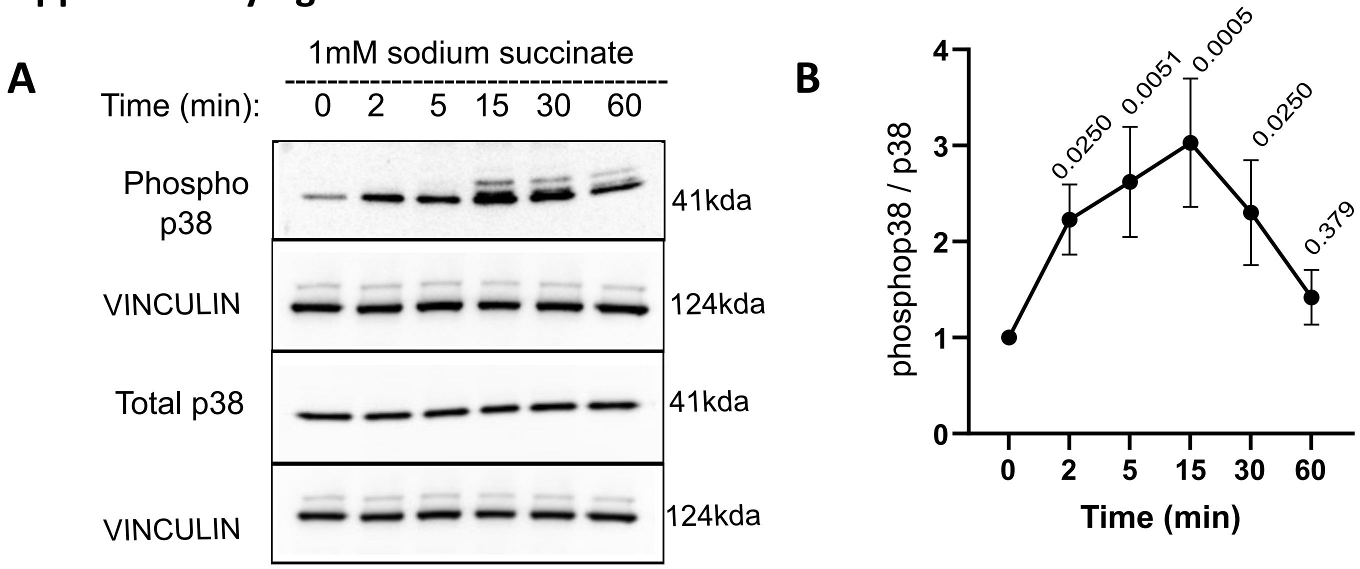

Supplementary figure 3: p38 activation in IPF fibroblasts treated with sodium succinate.
(A) Western blot for phospho p38 and total p38 with protein lysates from IPF patient-derived lung fibroblasts treated with 1mM sodium succinate over a time course of 60 minutes. Vinculin was used as loading control.
(B) Quantification of western blot in A.
Statistical analysis were performed using One-way ANOVA for repeated measures, followed by Holm-sidak test for multiple comparisons..

References.

1. **Singh S, Adam M, Matkar PN, Bugyei-Twum A, Desjardins J-F, Chen HH, Nguyen H, Bazinet H, Michels D, Liu Z, Mebrahtu E, Esene L, Joseph J, Ehsan M, Qadura M, Connelly KA, Leong-Poi H, and Singh KK**. Endothelial-specific Loss of IFT88 Promotes Endothelial-to-Mesenchymal Transition and Exacerbates Bleomycin-induced Pulmonary Fibrosis. *Scientific reports* 10: 4466, 2020.
2. **Miao L, Qi J, Zhao Q, Wu Q-N, Wei D-L, Wei X-L, Liu J, Chen J, Zeng Z-L, Ju H-Q, Luo H-y, and Xu R-H**. Targeting the STING pathway in tumor-associated macrophages regulates innate immune sensing of gastric cancer cells. *Theranostics* 10: 498-515, 2020.
3. **Atallah R, Gindlhuber J, Platzer W, Bärnthaler T, Tatzl E, Toller W, Strutz J, Rittchen S, Luschnig P, Birner-Gruenberger R, Wadsack C, and Heinemann A**. SUCNR1 Is Expressed in Human Placenta and Mediates Angiogenesis: Significance in Gestational Diabetes. *International journal of molecular sciences* 22: 2021.
4. **Li S, Song Y, Quach C, Guo H, Jang G-B, Maazi H, Zhao S, Sands NA, Liu Q, In GK, Peng D, Yuan W, Machida K, Yu M, Akbari O, Hagiya A, Yang Y, Punj V, Tang L, and Liang C**. Transcriptional regulation of autophagy-lysosomal function in BRAF-driven melanoma progression and chemoresistance. *Nature Communications* 10: 1693, 2019.
5. **Holle AW, Tang X, Vijayraghavan D, Vincent LG, Fuhrmann A, Choi YS, del Álamo JC, and Engler AJ**. In situ mechanotransduction via vinculin regulates stem cell differentiation. *Stem Cells* 31: 2467-2477, 2013.
6. **Byron A, Griffith BGC, Herrero A, Loftus AEP, Koeleman ES, Kogerman L, Dawson JC, McGivern N, Culley J, Grimes GR, Serrels B, von Kriegsheim A, Brunton VG, and Frame MC**. Characterisation of a nucleo-adhesome. *Nature Communications* 13: 3053, 2022.
7. **Merrill NM, Schipper JL, Karnes JB, Kauffman AL, Martin KR, and MacKeigan JP**. PI3K-C2α knockdown decreases autophagy and maturation of endocytic vesicles. *PLOS ONE* 12: e0184909, 2017.
